# Estimation of Duloxetine Hydrochloride in Pharmaceutical Formulations by RP-HPLC Method

**DOI:** 10.4103/0250-474X.49136

**Published:** 2008

**Authors:** Sejal K. Patel, N. J. Patel, K. M. Patel, P. U. Patel, B. H. Patel

**Affiliations:** S. K. Patel college of Pharmaceutical Education and Research, Ganpat University, Kherava-382 711, India

**Keywords:** Duloxetine HCl, isocratic mode, RP-HPLC, SSNRI

## Abstract

Simple, specific, accurate and precise method, namely, reverse phase high performance liquid chromatography was developed for estimation of duloxetine HCl in pharmaceutical formulations. For the high performance liquid chromatography method, Phenomenox C-18, 5 μm column consisting of 250×4.6 mm i.d. in isocratic mode, with mobile phase containing 0.01M 5.5 pH phosphate buffer: acetonitrile (60:40 v/v) and final pH adjust to 5.5±0.02 with phosphoric acid was used. The flow rate was 1.2 ml/min and effluent was monitored at 231 nm. The retention time was 5.61 min. The method was validated in terms of linearity, accuracy and precision. The linearity curve was found to be linear over 0.25-4 μg/ml. The limit of detection and limit of quantification were found to be 0.10 and 0.25 μg/ml respectively. The proposed method was successfully used to determine the drug content of marketed formulations.

Duloxetine HCl (DLX) is chemically, 2(+)-(S)-N-methyl-(gamma)-(1-naphthyloxy)-2 thiophenepropylamine hydrochloride^1^. Duloxetine hydrochloride is a newer selective serotonin and norepinephrine reuptake inhibitor (SSNRI) used for major depressive disorders^2^,^3^. Duloxetine is not official in any pharmacopoeia. A few methods in literature were reported for the determination of DLX and its key intermediate, desmethyl duloxetine in human serum by HPLC method^4^–^5^. Literature reported the characterization of phenolic impurities in duloxetine HCl samples by MS, NMR, X-ray-analysis^6^ and impurities formed by interaction of duloxetine HCl with various enteric polymers^7^. Simple UV Spectrophotometric method for estimation of duloxetine in formulation^8^ is reported but calibration range is from 5-50 μg/ml that shows the method is less sensitive. The present investigation describes a simple, rapid and reproducible RP-HPLC method with a calibration range of 0.25-4 μg/ml for duloxetine HCl in capsule dosage form that is more appropriate for routine analysis.

A Shimadzu's HPLC (LC-10AT vp) equipped with UV/Vis detector and manual injector of 20 μl loop, CP224S analytical balance (Sartorius), Ultrasonic bath (Frontline ultrasonic) were used for experiment. Volumetric flasks of 5, 10, and 50 ml capacity and other glassware (Borosil) were used.

Duloxetine hydrochloride (Torrent Pharmaceuticals Ltd. Ahmedabad, India), acetonitrile, methanol (HPLC grade, S. D. Fine Chemicals), triple distilled water, nylon 0.45 μm-47 mm membrane filter (Gelman laboratory, Mumbai, India), potassium dihydrogenphosphate, disodium hydrogen phosphate and phosphoric acid (AR grade, S. D. Fine Chemicals) were used for the study.

The standard stock solution of duloxetine HCl was prepared by dissolving 5 mg of drug in 50 ml volumetric flask separately using methanol. Final working standard solution of 25 μg/ml of duloxetine HCl was prepared by diluting 2.5 ml of the above solution to 10 ml with methanol. 0.25, 0.50, 0.75, 1.0, 2.0, 3.0 and 4.0 μg/ml concentration of solutions were prepared and injected under operating chromatographic conditions. Calibration curves were constructed by plotting peak area versus concentration of duloxetine HCl and the regression equation were calculated.

Assay of three different marketed products were performed. Twenty capsules were separately weighed and a quantity of mixed contents of 20 capsules equivalent to 5 mg of duloxetine hydrochloride was dissolved in methanol to obtain 1 mg/ml concentration, ultrasonicated and filtered through 0.45 mm cellulose nitrate filter. The solution was subjected to analysis as described earlier after suitable dilution. From the peak area of duloxetine HCl the amount of drug in the sample was computed using regression equation.

To optimize the HPLC parameters, several mobile phase compositions were tried. Satisfactory peak symmetry was obtained with mobile phase consisting of phosphate buffer (5.5 pH):acetonitrile (60:40 v/v) and final pH adjusted to 5.5±0.02 with phosphoric acid. Quantification was achieved with UV detection at 231 nm based on peak area. A representative chromatogram is shown in fig. 1. Parameters of chromatogram are shown in Table 1.

**Fig. 1:**
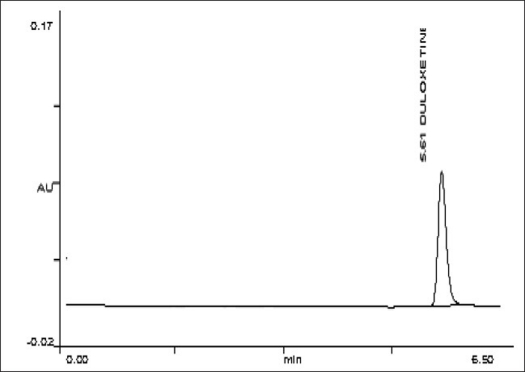
Chromatogram of duloxetine hydrochloride HPLC peak showing a retention time of 5.61 corresponding to duloxetine at λ 231 nm

**TABLE 1:** PARAMETERS OF CHROMATOGRAM

Parameters	Result
Linearity range	0.25-4 μg/ml
Correlation co-efficient	0.9994
Retention time	5.61 min.
Tailing factor	1.30
Asymmetry	1.44
Precision (% CV)	
Repeatability	0.512%
Intra day	0.37-1.16%
Inter day	1.13-1.94%
Accuracy	97.8-100.5%
Limit of detection	0.10 μg/ml
Limit of quantification	0.25 μg/ml

The regression data showed a good linear relationship over a concentration range of 0.25-4 μg/ml. The limit of detection and limit of quantification were found to be 0.10 and 0.25 μg/ml, respectively. As per the USP XXIII^9^, system suitability tests for HPLC were carried out on freshly prepared standard stock solution of duloxetine HCl and parameter obtained with 20 μl injection volume are summarized in Table 1. The intra-day and inter-day precision was determined by analyzing standard solutions in the concentration range of 0.25 to 4 μg/ml. The intra-day and inter-day precision results are given in Table 2. The results of the analysis of marketed formulations are well agreed with the label claim (Table 3). To study accuracy of the developed methods, recovery studies were carried out using standard addition method at four different levels for all the three brands and the % recovery was calculated (Table 4). The results revealed no interference from the excipients. The developed methods are simple, precise and accurate. The statistical data proved that methods are reproducible and selective for the analysis of duloxetine HCl in its marketed formulations.

**TABLE 2:** INTRADAY AND INTERDAY PRECISION DATA FOR DULOXETINE HYDROCHLORIDE AT 231NM

Conc. of duloxetine hydrochloride (μg/ml)	% CV (Intra day)	% CV (Interday)
0.25	1.16	1.94
0.50	0.58	1.22
0.75	0.60	1.13
1.0	0.54	1.16
2.0	0.40	1.15
3.0	0.37	1.21
4.0	0.38	1.16

**TABLE 3:** ESTIMATION OF DULOXETINE HYDROCHLORIDE IN CAPSULES

Formulation	Labeled amount of duloxetine hydrochloride (mg)	% Duloxetine hydrochloride found (n=3)
A	20	101.2±0.2
B	30	98.58±0.1
C	40	99.41±0.4

**TABLE 4:** ACCURACY DATA FOR DULOXETINE HYDROCHLORIDE AT 231NM

Formulation	Initial conc. (μg/ml)	Quantity of std. added (μg/ml)	Total quantity found Mean±SD	Accuracy ±% CV
A	0.5	0.5	0.978±0.028	97.8±1.86
	0.5	1.5	1.976±0.034	98.8±1.72
	0.5	2.5	3.015±0.048	100.5±1.59
	0.5	3.5	3.971±0.067	99.27±1.68
B	0.5	0.5	0.998±0.031	99.8±1.63
	0.5	1.5	1.959±0.032	97.95±1.74
	0.5	2.5	2.999±0.043	99.96±1.45
	0.5	3.5	4.012±0.053	100.3±1.63
C	0.5	0.5	1.002±0.025	100.2±1.24
	0.5	1.5	2.003±0.039	100.15±1.55
	0.5	2.5	3.021±0.052	100.7±1.79
	0.5	3.5	4.015±0.074	100.37±1.44
